# Outcome of acute East African trypanosomiasis in a Polish traveller treated with pentamidine

**DOI:** 10.1186/1471-2334-14-111

**Published:** 2014-02-27

**Authors:** Małgorzata Paul, Jerzy Stefaniak, Piotr Smuszkiewicz, Marjan Van Esbroeck, Dirk Geysen, Jan Clerinx

**Affiliations:** 1 Department and Clinic of Tropical and Parasitic Diseases, University of Medical Sciences, Przybyszewskiego 49, Poznań, Poland; 2 Department of Anaesthesiology, Intensive Therapy and Pain Treatment, University of Medical Sciences, Poznań, Poland; 3 Department of Clinical Sciences, Institute of Tropical Medicine, Antwerp, Belgium; 4 Department of Veterinary Sciences, Institute of Tropical Medicine, Antwerp, Belgium

**Keywords:** East African trypanosomiasis, *Trypanosoma brucei rhodesiense*, Imported tropical disease, Travel medicine, Pentamidine

## Abstract

**Background:**

African trypanosomiasis is a parasitic infection sporadically imported to Europe by tourists or immigrants returning from endemic areas. We present the first and an unusual case of East African trypanosomiasis imported to Poland by a patient returning from a tourist trip to Uganda and Rwanda, which was successfully treated with pentamidine.

**Case presentation:**

A 61-year-old Polish man was admitted to the Department because of high-grade fever and multi-organ dysfunction after a tourist trip to East Africa. He experienced a single tsetse fly bite during a safari trip to the Queen Elizabeth National Park in Uganda. On admission, his clinical status was severe, with high fever of 41ºC, preceded by chills, bleeding from the gums and oral mucosa, haemorrhages at the sites of venipuncture, numerous ecchymoses, fine-spotted skin rash, tachycardia, hepatosplenomegaly, dehydration, jaundice, dyspnoea, hypoxaemia, generalised oedema and oliguria. There was a typical non-painful trypanosomal chancre with central necrosis and peripheral erythema on his left arm. Laboratory investigations showed leucopenia, thrombocytopenia, haemolytic anaemia, hyperbilirubinaemia, hypoglycaemia, elevated creatinine and urea, high activity of aminotransferases, elevated levels of inflammatory markers, hypoproteinaemia, proteinuria, abnormal clotting and bleeding times, low fibrinogen level, metabolic acidosis, and electrolyte disturbances. A peripheral blood smear showed numerous *Trypanosoma brucei* trypomastigotes with a massive parasitaemia of 100,000/μl. *T. brucei rhodesiense* subspecies was finally identified on the basis of the characteristic serum resistance-associated gene using a polymerase chain reaction, and a seroconversion of specific immunoglobulin M and G antibodies in the peripheral blood by enzyme-linked immunosorbent assay. Serological tests for *T. brucei gambiense* subspecies were negative. A severe clinical course of acute rhodesiense trypanosomiasis with renal failure, respiratory distress, disseminated intravascular coagulation syndrome, haemolysis, liver insufficiency and myocarditis was confirmed. Intensive anti-parasitic and symptomatic treatment was immediately instituted, including intravenous pentamidine, plasmaphereses, oxygen therapy, blood transfusion, catecholamine administration, and fluid infusions, as well as haemostatic, hepatoprotective, anti-inflammatory, antipyretic and diuretic drugs. The final outcome was a full recovery with no late sequelae.

**Conclusion:**

Sleeping sickness should always be considered in the differential diagnosis of fever in people returning from safari trips to the national parks or nature reserves of sub-Saharan Africa.

## Background

Human African trypanosomiasis (HAT) is a protozoan, vector-borne infection occasionally imported to Europe by tourists, military personnel, expatriates and immigrants returning from endemic areas of sub-Saharan Africa. Only a handful of cases are reported every year in European tropical institutes [1,2]. A delay in medical diagnosis or inappropriate treatment of the disease, which is usually rarely considered in the differential diagnosis of fever in a patient returning from tropical areas, may lead to severe multi-organ injury or even a fatal clinical prognosis during an acute period of generalised parasitaemia of East African trypanosomiasis or in a chronic phase of meningoencephalitis in the West African form of the infection [3,4]. West African trypanosomiasis, which circulates in the human environment, is characterised by a more benign, slowly progressing and chronic clinical course of the disease. On the contrary, infection with *Trypanosoma brucei rhodesiense,* which is a pure zoonosis transmitted through bites of infected tsetse flies (*Glossina* spp.) that feed on cattle or ungulate wild animals, is characterised by a typical chancre at the bite site, followed by sudden onset, high grade fever 3 to 10 days thereafter, fulminant manifestatation of clinical symptoms and a poor prognosis. Usually there is rapid progression towards multi-organ disease, rendering appropriate treatment a medical emergency. In many instances, the recommended first line treatment with suramin is not readily available, and the lack of promptly instituted specific therapy may increase the risk of serious complications.

HAT caused by *T. b. gambiense,* imported to Europe by travellers, is more frequently observed in migrants and expatriates residing in rural endemic areas, while more severe *T. b. rhodesiense* infections are mainly reported in tourists and hunters returning from East African national parks and game ranches, where antelopes constitute an important reservoir of the protozoan.

Uganda is a unique country in the world, where *T. b. rhodesiense* (responsible for acute disease) and *T. b. gambiense* subspecies (causing the chronic form of the infection), both occur endemically. More disquietingly, however, areas affected by East African trypanosomiasis have significantly increased and continuously enlarged within the past few years, to include new districts and foci through repopulation and subsequent migration of the livestock reservoir of this neglected anthropozoonotic disease. Cattle are the main reservoir for *T. b. rhodesiense* in rural areas. By contrast, West African trypanosomiasis caused by *T. b. gambiense* has no documented epidemiologically significant animal reservoir in the natural environment. Suitable *Glossina* spp. vectors abound in the Queen Elizabeth National Park, frequently visited by foreign travellers as well, and the replenishment of wildlife has probably amplified the natural host reservoir of the parasite. Three species of the vector are currently responsible for the *T. b. rhodesiense* infection of humans in Uganda *Glossina fuscipes fuscipes, G. pallidipes and G. morsitans morsitans*, but only the first two constitute a source for parasite transmission in the Queen Elizabeth National Park. At present, West African trypanosomiasis is confined to the northern part of Uganda where *G. fuscipes fuscipes* predominates, but the dividing line between both species is narrowing [5-8].

In this study, we present the first and an unusual case of acute East African trypanosomiasis, imported to Poland by a patient returning from a tourist trip to Uganda and Rwanda, which was successfully treated with pentamidine. This is probably the first case of imported severe Rhodesian trypanosomiasis with extremely high intensity of infection and sensitivity to pentamidine described in the literature [1,9].

## Case presentation

On 28 July 2009, a 61-year-old Polish man, with no previous eventful medical history, was transferred as an emergency from a regional hospital in an air ambulance and admitted to the Department of Tropical Diseases because of high-grade fever and multi-organ dysfunction after a trip to East Africa. The patient spent 18 days on a package tourist trip to Uganda and Rwanda organised by a Polish travel agency and had returned 4 days prior to admission. During the travel, in a group of 16 other tourists, he visited the most famous national parks in Uganda and Rwanda, and had close contact with some wild animals (mainly gorillas and chimpanzees) but never participated in hunting. He did not comply with tropical hygiene measures; he frequently wore shorts and a T-shirt. He experienced a painful single tsetse fly bite on his left arm on 14 July 2009 during a safari trip to the Queen Elizabeth National Park in Uganda, but did not seek for medical advice from the local physician. No other person from the Polish tourist group was infected.

Before the trip to the tropics, the patient did not consult with any travel medicine specialist; however, he took antimalarial chemoprophylaxis in form of atovaquone and proguanil (Malarone) regularly, and was vaccinated against selected infectious diseases in a regional sanitary-epidemiological centre (yellow fever, tetanus, viral hepatitis A and B). For 10 years he had been travelling to various tropical and subtropical countries, including those of Africa, Latin America, Southeast Asia and the Far East islands of Indonesia. He had experienced multiple insect bites in the tropics, but had never visited a specialist in travel medicine or tropical diseases.

Six days after a whole day spent at the Queen Elizabeth National Park in Uganda, a painless skin lesion appeared on the left arm, at the site of the tsetse fly bite. Two days later, he had a high fever of 40°C proceeded by chills, together with weakness, fatigue and malaise. After arrival in Poland on 24 July 2009, he went directly to his family doctor and was treated with a beta-lactam antibiotic for 4 days without any improvement. On the 7th day of illnesses, after symptoms of multi-organ injury had begun, he was admitted to a regional infectious diseases hospital in South-East Poland, and then transferred to the Department of Tropical and Parasitic Diseases in Poznań with the suspicion of complicated malaria, viral haemorrhagic fever or bacterial sepsis.

**Figure 1:**
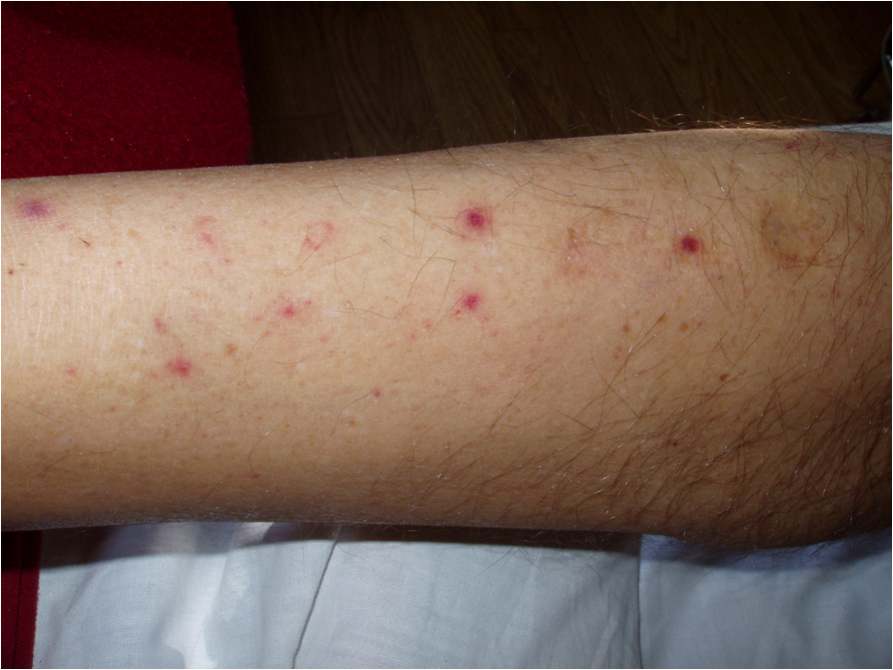
**Numerous ecchymoses on the skin of the lower extremities in a patient infected with *T. b. rhodesiense*.**

**Figure 2:**
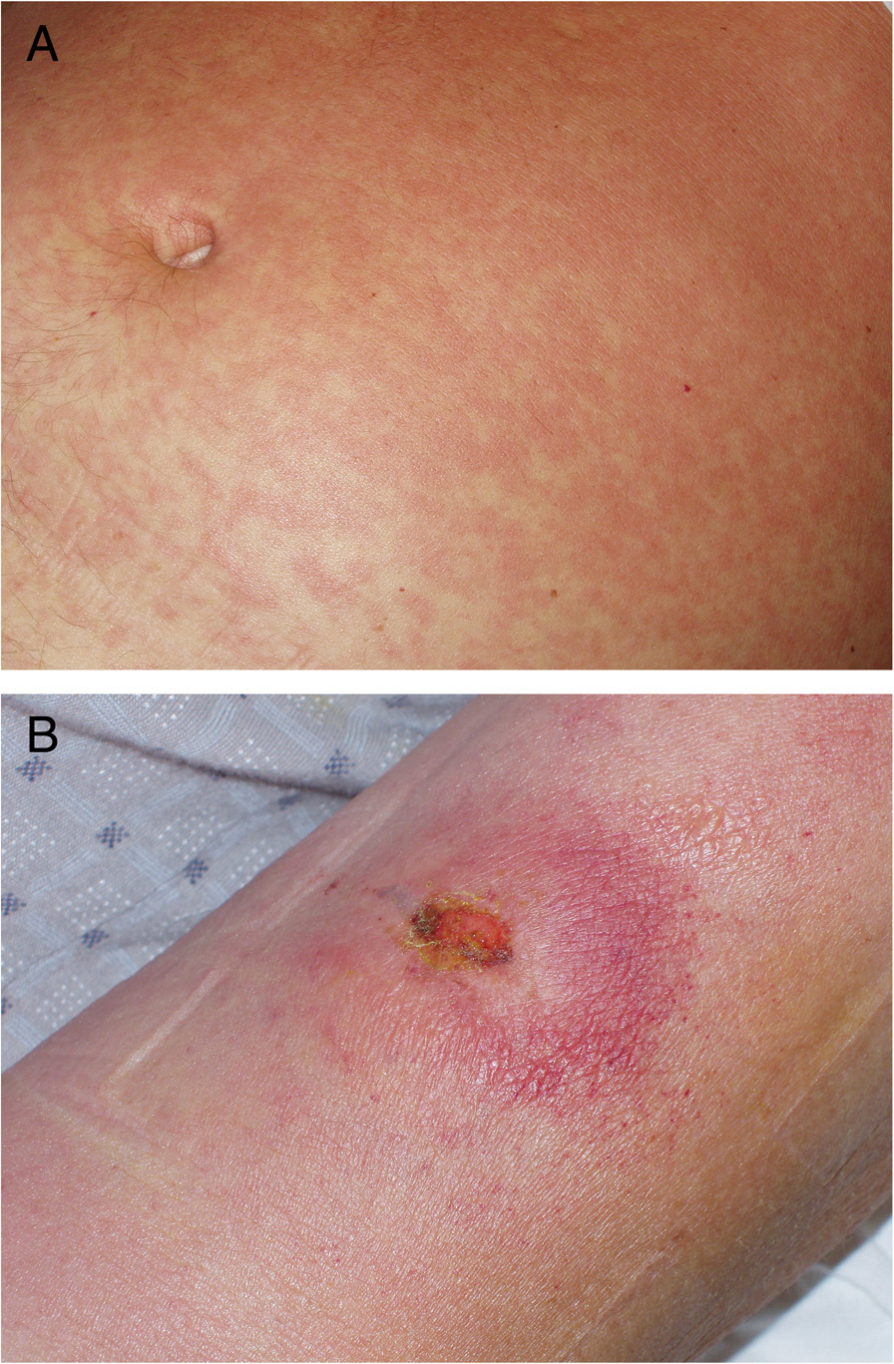
Typical clinical manifestations of acute African trypanosomiasis imported from Uganda: A) Fine-spotted pink rash on the skin of the abdomen (”trypanid rash”); B) Trypanosomal chancre on the skin of the left arm.

On admission, on the 8th day of clinical symptoms, the clinical status of the patient was severe, with a fever of 41°C, chills, numerous ecchymoses on the lower extremities, petechiae on a large area of the skin of the abdomen and chest, fine-spotted non-pruritic skin rash, tachycardia of 124/min, hepatosplenomegaly, signs of dehydration, prostration, jaundice, dyspnoea, hypoxaemia (oxygen saturation 78.9%), generalised oedema and oliguria (urine output 250 ml). Most worrying were signs of bleeding from the gums and oral mucosa and haemorrhages at the sites of venipuncture, suggestive of disseminated intravascular coagulation (DIC) syndrome (Figures 1 and 2A). The patient was conscious and very well orientated (15 points in Glasgow Coma Scale) but seemed to respond rather slowly to commands. On his left arm, there was still a visible, typical, painless trypanosomal chancre of 6 cm in diameter with a depressed dark centre surrounded by peripheral erythema (Figure 2B).

Routine laboratory examinations showed leucopenia (2.09 G/l), severe thrombocytopenia (10 G/l), shift to the left in Schilling’s blood count (band cells 25%, segmented cells 48%), lymphopenia (12%), hyperbilirubinaemia (2.37 mg/dl), hypoglycaemia (30 mg/dl), elevated concentrations of creatinine (2.92 mg/dl) and urea (93 mg/dl), high activity of the liver enzymes (alanine aminotransferase 208 U/l, aspartate aminotransferase 217 U/l, gamma-glutamyl transpeptidase 162 U/l), marked elevation of the inflammatory markers (C-reactive protein 221 mg/l, procalcitonin 3.22 ng/ml), hypoproteinaemia (6.1 g/dl), proteinuria, presence of hyaline casts in the urine, abnormal clotting and bleeding times (international normalisation ratio 1.34, activated partial thromboplastin time 55 s, prothrombin time 16.2 s), low level of fibrinogen (173 mg/dl) and other parameters consistent with DIC syndrome (D-dimers > 35.85 mg/l, antithrombin III [ATIII] 61%), metabolic acidosis (lactic acid 6.5 mmol/l, sO_2_ 65.8%, pH 7.286, pO_2_ 38.8 mm Hg, pCO_2_ 41.9 mm Hg, HCO_3_ 19.3 mmol/l, BE - 6.7 mmol/l), and electrolyte disturbances (Na 137 mmol/l, K 3.09 mmol/l) (Table 1).

**Table 1:** Clinical and paraclinical features during hospitalisation and follow-up of a patient with African trypanosomiasis

	**D0**	**D3**	**D7**	**D15**	**D30**	**D60**	**D90**	**D 120**
	**28-7**	**31-7**	**3-8**	**13-8**	**2-9**	**30-9**	**28-10**	**8-12**
Inoculation chancre	+	+	+	+	+/−	-	-	-
Fever	+	+	-	-	-	-	-	-
Anaemia	-	+	+	+	+	-	-	-
Jaundice	+	+	+	-	-	-	-	-
Splenomegaly	+	+	+	-	-	-	-	-
Spontaneous bleeding	+	+	-	-	-	-	-	-
Respiratory distress	+	+	-	-	-	-	-	-
Renal injury	+	+	-	-	-	-	-	-
Hgb (g/dl)	14.5	13.8	10.0	9.2	10.6	15.1	16.4	16.2
Thrombocytes (n/μl)	10000	9000	70000	445000	370000	216000	206000	216000
Parasitaemia (n/μl)	100000	0	0	0	0	0	0	0
ALT (IU/l)	208	362	68	18	20	30	34	29
AST (IU/l)	217	237	48	12	12	20	21	20
Bilirubin (total, mg/dl)	2.37	3.48	4.52	0.76	0.60	0.55	0.68	0.75
Fibrinogen (mg/dl)	173	125	136	256	276	281	274	278
Creatinine (mg/dl)	2.92	1.71	0.57	0.46	0.70	0.91	0.96	0.96
Tbgam IFAT	-		-	-	-	-	-	-
Tbgam CATT	-		-	-	-	-	-	-
Tbrhod ETat 1:1	-		+	+	+	-	-	-
Tbrhod ETat 1:18	-		-	-	-	-	-	-
Total serum IgM (mg/l)	1610		3530	7030	5050	3370	3120	2170
T.brucei complex PCR-RFLP	+							
T.b.rhod. PCR SLG polymorphism	+							
T.b.rhod PCR SRA	+ (287 bp)							

*Abbreviations*: *Hgb* hemoglobin, *ALT* alanine aminotransferase, *AST* aspartate aminotransferase, *Tbgam Trypanosoma brucei gambiense*, *Tbrhod Trypanosoma brucei rhodesiense*, *CATT* Card Agglutination Test for Trypanosomiasis, *IFAT* Indirect Fluorescent Antibody Test, *ETat* Edinburgh Trypanosoma Antigenic Type, *PCR* Polymerase Chain Reaction, *RFLP* Restriction Fragment Length Polymorphism, *SLG* Spliced Leader Gene Polymorphism Marker, *SRA* Serum Resistance-Associated Gene, *“-“* negative result, *“+”* positive result, bp: base pair.

**Figure 3:**
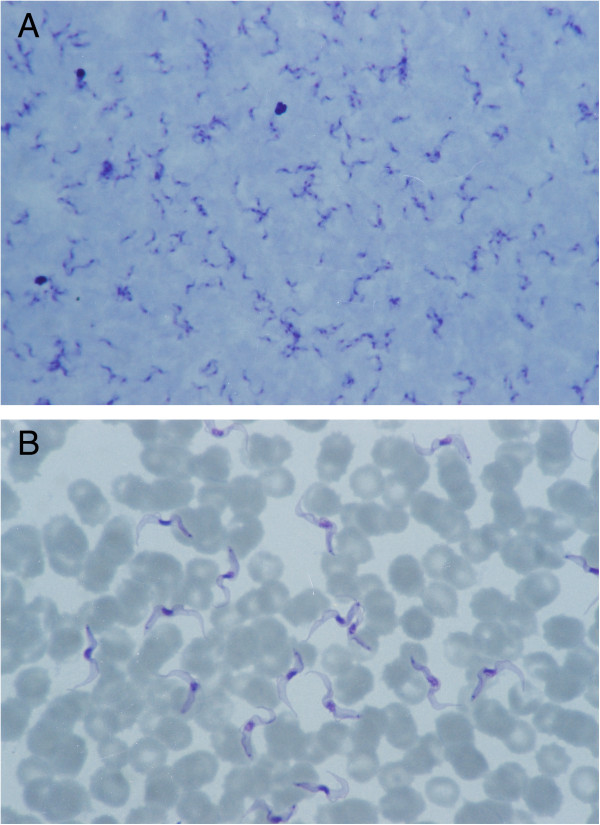
**Trypomastigotes of *T. b. rhodesiense *** **in the peripheral blood stained with Giemsa: A) Thick blood film. Magn. 400×; B) Thin blood film. Magn. 1000x.**

On admission, the peripheral blood smear showed numerous *Trypanosoma brucei* trypomastigotes, with a massive parasitaemia of 100,000 per 1 μl using the Carpentier’s cell or an average of 45 protozoans in a higher magnification microscopic field of 1000×, and 116 parasites in a lower magnification of 400× in direct microscopy (Figure 3).

Electrocardiography showed negative T wave in aVR and precordial leads V2-V6 with sinus tachycardia and pathological left-axis deviation. There was no sign of bleeding to the central nervous system as was demonstrated in a computerised tomography scan, or to the retina on ophthalmoscopic examination. Lumbar puncture showed no trypomastigotes in the precipitate of the cerebrospinal fluid after centrifugation; leucocyte count (3 cells/μl) and protein concentration (266 mg/l) were normal.

Because of the severe clinical condition of the patient, and the non-availability of suramin on the day of admission, intensive anti-parasitic treatment with intravenous pentamidine isethionate (Pentacarinat, Sanofi-Aventis, Frankfurt am Main, Germany) at a dose of 300 mg every 2 days for 14 days (7 doses) was immediately instituted. At the same time, symptomatic treatment was commenced, which included passive oxygen therapy, repeated transfusions of blood (2 units), plasma (9 units), platelets (50 units), antithrombin III (500 units) and 20% albumins (400 ml), fluid infusions, as well as the administration of haemostatic, hepatoprotective, anti-inflammatory, antipyretic and diuretic drugs. Because of respiratory distress, the patient was monitored in an intensive care unit for 6 days.

Treatment was well tolerated and led to a rapid parasite clearance within 72 hours, after the 2nd dose of pentamidine. Renal impairment and electrolyte imbalance did not require haemodialysis, but we opted for four cycles of plasmapheresis, starting on the day of the extinction of all trypomastigotes from the peripheral blood. The complete destruction of the circulating trypanosomes and the massive release of antigens caused a single incidence of hypotension (blood pressure 90/55 mm Hg) and the patient required an additional dose of catecholamines (norepinephrine). In spite of the transfusions, the platelet count was lowest on the 2nd day (3 G/l), and other laboratory parameters of DIC syndrome were most evident on the 4th day of hospitalisation (fibrinogen 108 mg/dl, ATIII 55%). Haemolytic anaemia was noticed on the 3rd day of treatment and was most severe on the 10th day after admission (Hgb 7.8 g/dl, RBC 2.51 T/l, Hct 23.2%) with an increase in bilirubin level of up to 5.75 mg/dl. The fever subsided on the 4th day of anti-trypanosomal therapy. Normal renal function was restored on the 6th day, signs of respiratory distress receded on the 7th day, coagulopathy ceased on the 8th day, and liver enzymes were normalised on the 10th day of hospitalisation. The electrocardiographic changes seen on admission normalised during treatment.

The final outcome of the intensive therapy was that of success, and the patient was discharged home after 3 weeks, without any residual signs of organ dysfunction. The patient has been asymptomatic since then, and there have been no signs of disease persistence or reactivation during the 3 years of clinical and laboratory follow-up.

### Diagnostic differentiation of *Trypanosoma brucei* complex

Molecular diagnosis of East African trypanosomiasis was done retrospectively after anti-parasitic therapy, using a two-step test on the pre-treatment whole blood sample containing trypanosomes (Institute of Tropical Medicine, Antwerp, Belgium). In the first step, the presence of *Trypanosoma brucei* complex was confirmed by means of a polymerase chain reaction - restriction fragment length polymorphism (PCR-RFLP) using the small subunit rDNA marker [10]. In the subsequent step, a specific in-house PCR-based test to identify *T. b. rhodesiense* using the spliced leader gene polymorphism marker was carried out and proved to produce a positive response. In addition, a PCR based test for the serum resistance-associated (SRA) gene specific for *T. b. rhodesiense* showed a positive result*.*

Antibody tests for *Trypanosoma brucei gambiense* and *rhodesiense* were carried out using card agglutination test for trypanosomiasis (CATT), indirect fluorescent antibody test (IFAT), and enzyme-linked immunosorbent assay (ELISA) in two different dilutions. The serological tests, which are produced under full quality control, are currently manufactured only at the Institute of Tropical Medicine in Antwerp (Belgium) from where field kits containing reagents, control sera, and test accessories can be obtained (http://www.itg.be/itg/GeneralSite/default.aspx?WPID=471&MIID=433&L=E; contact Philippe Büscher; pbuscher@itg.be). A positive reaction for *T. b. rhodesiense* subspecies was observed in the serum samples (Table 1).

## Discussion

We presented the first case of sleeping sickness imported to Poland, and the first case of documented *Trypanosoma brucei* infection in a tourist from Central-Eastern Europe. Moreover, this was the first recorded case in the past 25 years of acute-stage East African trypanosomiasis occurring in a European traveller infected in Uganda (Eastern Africa).

Although East African trypanosomiasis is hyperendemic in the south-eastern part of Uganda, it has spread to the western and central parts of the country during the last decade, and this is partially due to the movement of cattle and their local trading to these regions from the original infection belt. In fact, HAT has become a re-emerging parasitic infection of public health concern in Uganda, where the geographical distribution of tsetse fly-affected areas has extended to previously uninfected provinces [5-8,11]. According to the National Sleeping Sickness Control Program in Uganda, more than 2,500 presumed *T. b. rhodesiense* cases have been reported since 2000, and mortality rates are higher in recently affected districts. The disease has a tendency to a seasonal transmission with the highest risk from January to March, as spreading of infection is mostly related to livestock migration and cattle trading [12].

Among all HAT cases imported to Europe since 2005, most of them were expatriates or immigrants returning from sub-Saharan Africa; but during the last years an increasing number of sleeping sickness has been reported in tourists [1,13-16]. Over the last decade, non-endemic human infections with *T. brucei* in European travellers have been reported mainly from Tanzania, Gabon, Zambia, Angola, Guinea, Malawi and Central African Republic. So far, imported HAT cases have been registered in France, Italy, Spain, the United Kingdom, Belgium, Germany, the Netherlands, Switzerland and Scandinavia [1,2,17-24].

The presented patient did not comply with any tropical hygiene measures, and wore inappropriate clothing in areas at risk. European tourists travelling to destinations with a significant risk of HAT, such as national parks, nature reserves and safari camps, should be instructed on preventive measures against tsetse bites, particularly wearing long sleeve shirts and trousers made of a thick cotton material, without bright or contrasting colours, impregnated with permethrin, as well as regular application of insect repellents. If skin symptoms occur, consultation with an experienced travel medicine clinician should be considered as a matter of urgency, in order to reduce the high fatality rate if generalised signs of HAT develop. As in the case of our patient, an inoculation chancre is typically present in nearly all reported patients with imported HAT [1,18,20,22-24], and it is a clinical sign of utmost importance in guiding the differential diagnosis [25]. It may be mistaken for a rickettsial chancre by less experienced health practitioners or simply overlooked with potentially disastrous consequences [4]. According to Odiit and colleagues, most HAT cases that originated from epidemic areas in Uganda were not diagnosed by the national health care system, including fatal cases, despite their contact with medical facilities [26].

Early diagnosis and prompt administration of effective anti-parasitic treatment are the key prognostic factors in a patient’s clinical prognosis and increase the probability of survival. In HAT caused by *T. b. rhodesiense*, the disease has a tendency of rapid progression to the stage of central nervous system involvement between 3 weeks to 2 months after a primary infection. Most HAT deaths are reported within the first 6 months of clinical manifestations [27-29].

The role of the geographical location, parasite genotype and specific host inflammatory cytokine response in the clinical expression of the disease in humans has been studied by many authors. Currently, an analysis of some genetic markers of *T. b. rhodesiense* virulence seems to be the best option for a predictive evaluation of HAT cases, and a practical determination of clinical course severity. MacLean et al. reported that infections with the Tororo genotype were characterised by an increased probability of disease progression and a rapid development of the meningoencephalitic stage, and higher plasma interferon gamma levels, while the Soroti genotype was well correlated with a milder clinical course of HAT cases [28]. Similarly, severe clinical status of HAT patients and its rapid progression to meningoencephalitis in *T. b. rhodesiense* infections from Uganda was related to elevated concentrations of tumour necrosis factor alpha (TNF-alpha) in serum samples. On the contrary, more benign infections with *T. b. rhodesiense* in Malawi were associated with high levels of transforming growth factor beta but not TNF-alpha activation [29].

In the reported case, circulating trypomastigotes were easily identified in the peripheral blood samples collected on admission at the emergency room, and stained by Giemsa. The short incubation period of the infection and the severe clinical status of the patient, accompanied by multi-organ injury, suggested an acute Rhodesian form of HAT caused by a highly pathogenic strain. Molecular differentiation of *T. brucei* complex by finding the characteristic SRA gene and S-locus glycoprotein gene polymorphism marker of *T. b. rhodesiense* subspecies, in whole blood collected before treatment, using a PCR technique, considerably improved the diagnostic accuracy and finally confirmed the clinical diagnosis (Table 1).

The SRA gene, first isolated from a Ugandan strain of *T. b. rhodesiense,* has been shown to be a marker of the parasite invasiveness for humans. This gene has also been identified in several other isolates of *T. b. rhodesiense* from Ethiopia, Kenya, Tanzania, Zambia and Botswana, but not in the other human pathogenic trypanosomes in Africa, including *T. b. gambiense*[13,30]. Further analysis showed a significant SRA gene polymorphism and differences in immune response profiles in host populations from variable geographical areas [29]. Strain typing using microsatellite markers has proven the molecular heterogeneity between different *T. b. rhodesiense* isolates, which can be related to considerable differences in the degree of infectivity to human beings according to genetic variants [28].

Serological diagnosis, particularly the CATT widely used in the field by medical officers, is more helpful in *T. b. gambiense* infections, when trypomastigotes may be difficult to be found in the peripheral blood or lymph. Home-made IFAT or ELISA performed in scientific laboratories is very useful for the differential diagnosis between both subspecies. In the case described above, we detected a seroconversion of immunoglobulin (Ig) M and IgG specific antibodies in the peripheral blood by ELISA on the 7th day of hospitalisation or 15th day of clinical symptoms. Total IgM reached the highest level on the 7th day of treatment and persisted longer than *Trypanosoma*-specific immunoglobulins. Serological tests for *T. b. gambiense* subspecies (CATT, IFAT, ELISA) were all negative, confirming the final diagnosis (Table 1).

Pentamidine is recommended to be the standard therapy for acute West African trypanosomiasis. Suramin is generally considered as the drug of choice for an early stage of Rhodesian sleeping sickness; melarsoprol alone or in a combination with nifurtimox is proposed for a late stage of the illness [3,13,31,32]. A combined therapy with suramin and eflornithine seems to be very promising [32,33]. In case of unavailability of suramin, treatment with pentamidine plays a crucial role in the prevention of severe complications of second-stage HAT, characterised by poor clinical prognosis [1]. In a large study of 56 travellers from non-endemic countries infected with *T. b. rhodesiense*, 7% were treated with pentamidine alone [34]. In some other patients with early stage of Rhodesian sleeping sickness, anti-protozoan therapy with pentamidine has been initiated and then switched to suramin upon availability [15]. Therefore, pentamidine is accepted to be an alternative drug for the management of an early phase of *T. b. rhodesiense* infection [35].

Among all imported cases of East African trypanosomiasis reported in the literature, this was also the first documented patient successfully treated with pentamidine instead of suramin, despite the critically high parasitaemia, which is extremely rarely observed. After the administration of suramin, the blood stage trypomastigotes disappeared within 3 days in all recently imported cases [13,16]. This was finally observed in this patient treated with pentamidine alone. Following the elimination of parasitaemia, clinical recovery occurred fairly rapidly. Clinical symptoms and laboratory parameters of multi-organ injury resolved within 10 days, without late complications. There have been no signs of disease persistence or relapse during the 3 years of clinical follow-up.

Patients should usually be followed-up for 3 months (acute haematogenous stage) or for up to 2 years (meningoencephalitic stage), to confirm complete eradication. There is a risk of the chronic suppression of the disease in improperly treated patients [32]. In this case, anti - *T. b. rhodesiense* IgM-IgG antibodies were observed in serum samples for 4 weeks and were later not detectable. Total IgM lasted longer, but after significantly rising 1 week after admission to hospital, it tended to diminish during the follow-up period (Table 1). During serological monitoring, serum IgM levels may actually increase before they finally decrease, and should not be regarded as a sign of treatment failure. Serum antibodies may appear well after the commencement of treatment, and diminish over time with the elimination of parasites. A 2-fold reduction in serum antibody titre may be interpreted as a sign of at least temporary parasite reduction, but it cannot be used to ascertain definitive cure [36]. Further analysis of some genetic markers of sensitivity and/or resistance to pentamidine in *T. b. rhodesiense* strains from Uganda has been promising.

## Conclusions

We recommend that proper health education on threats related to contact with tsetse flies and the possibilities of their prevention is important for persons travelling to areas with a high risk of *T. brucei* infection in Africa. Sleeping sickness should always be considered in the differential diagnosis of fever in people returning from safari trips to national parks or nature reserves of sub-Saharan Africa. Imported African trypanosomiasis should be treated in reference centres of tropical diseases or travel medicine, which have good clinical experience in rare exotic infections and direct access to intensive care facilities. Finally, in *T. b. rhodesiense* infections without central nervous system involvement, treatment with pentamidine may be considered an option until access to suramin has been achieved.

### Consent

Written informed consent was obtained from the patient for the publication of this case report and any accompanying images. A copy of the written consent is available for review by the Editor of this journal.

## Competing interests

The authors declare that they have no competing interests.

## Authors’ contributions

MP was directly responsible for the patient, performed the clinical and parasitological examinations and was a major contributor in writing the case report. PS coordinated intensive care monitoring. ME carried out the molecular biology tests. DG was responsible for the immunoassays. JC conceived the study, and participated in its design and coordination and helped to draft the manuscript. JS reviewed the paper. All the authors read and approved the final version of the manuscript.

## Pre-publication history

The pre-publication history for this paper can be accessed here:

http://www.biomedcentral.com/1471-2334/14/111/prepub
